# Characterization of feline nonsteroidal anti-inflammatory drug activated gene-1 *(fNAG-1)* and its protective function in kidney cells

**DOI:** 10.1186/s12917-025-04781-1

**Published:** 2025-05-21

**Authors:** Pattawika Lertpatipanpong, Hyunjin Moon, Jung Eun Seo, Minsu Kim, Seung Joon Baek

**Affiliations:** 1 Laboratory of Signal Transduction, College of Veterinary Medicine, Research Institute for Veterinary Science, Seoul National University, 1 Gwanak-ro, Gwanak-gu, Seoul, 08826 Republic of Korea; 2 Department of Pharmacy Practice, Faculty of Pharmaceutical Sciences, Naresuan University, Phitsanulok, 65000 Thailand; 3 Center for Veterinary Integrative Medicine, College of Veterinary Medicine, Seoul National University, Seoul, 08826 Republic of Korea

**Keywords:** NAG-1, Phytochemicals, Kidney, Obesity, Cat, Mitochondria

## Abstract

**Background:**

Domestic cats are susceptible to obesity and chronic renal failure, leading to significant health risks. Nonsteroidal anti-inflammatory drug-activated gene (NAG-1), also known as growth differentiation factor 15 (GDF15), is a member of the transforming growth factor-β superfamily and has been associated with anti-obesity properties and preservation of kidney function. While the NAG-1 sequence has been extensively studied in several species, a comprehensive understanding of feline NAG-1 remains limited. This study aimed to investigate the nucleotide sequence of feline NAG-1 and its biological role in kidney protection through in-vitro experiments.

**Methods:**

The feline *NAG-1* cDNA was isolated from the feline uterus, and its sequence was analyzed and compared to sequences from other species, including humans. Expression patterns of feline NAG-1 in various tissues, particularly the liver and kidney, were determined. Furthermore, the effects of different phytochemicals and NSAIDs known to induce NAG-1 expression were assessed using Crandell-Rees Feline Kidney (CRFK) cells.

**Results:**

The analysis revealed that feline NAG-1 shares similarities with human NAG-1 and exhibits high expression levels in the liver and kidney of cats. Treatment with tolfenamic acid, quercetin, and resveratrol significantly increased NAG-1 expression in CRFK cells. Subsequently, CRFK cells overexpressing feline NAG-1 were utilized to investigate the functional roles of NAG-1 in feline kidney health. High-content screening analysis demonstrated that NAG-1 overexpression in cat kidney cells enhanced mitochondrial membrane potential, reduced reactive oxygen species (ROS) generation in both whole cells and mitochondria, and downregulated the expression of Bax, a pro-apoptotic protein, under conditions of ROS-induced stress. These findings indicate the renoprotective role of NAG-1.

**Conclusion:**

This study highlights the significant role of NAG-1 in feline kidney cells, revealing its high expression in the liver and kidney and demonstrating its protective effects on kidney function. These results underscore the potential of NAG-1 as a key factor in kidney protection. Future research should focus on further elucidating the molecular pathways involved and exploring therapeutic strategies to harness NAG-1 for managing obesity-related renal dysfunction in cats.

**Supplementary Information:**

The online version contains supplementary material available at 10.1186/s12917-025-04781-1.

## Introduction

The rise in life expectancy in cats, similar to what has been observed in humans, has led to a higher incidence of kidney diseases such as hydronephrosis, renal failure, nephrolithiasis, renal calcification, renal amyloidosis, and glomerulonephritis [1–3]. Since there is no cure for kidney disease in cats, efforts focus on delaying the progression of chronic kidney disease (CKD) and improving quality of life [4]. Obesity in cats is a significant risk factor for overall health and has been linked to various disorders. Similar to humans, obese cats often exhibit signs of insulin resistance [5, 6]. In human, both obesity and insulin resistance are potential risk factors for accelerating the development and worsening progression of kidney disease, which may lead to shortened life span [7]. However, evidence connecting obesity to CKD in cats remains limited. Beyond being a serious health issue for both cats and humans, obesity is also a key animal welfare concern. Preventing or delaying the onset of obesity-related conditions is crucial for feline health, and dietary factors have been proposed to play a role in preventing CKD in cats [8]. Therefore, identifying a new potential biomarker for obesity is crucial for advancing our understanding of the underlying mechanisms of this complex disease, which could ultimately lead to the development of new and improved treatments for both cats and humans.

Nonsteroidal anti-inflammatory drug-activated gene (NAG-1) is a transforming growth factor-beta superfamily gene identified as a multi-functional cytokine [9]. NAG-1 is synthesized in the cytoplasm and secreted into the extracellular matrix in two different forms: mature and pro-form [10]. The mature form is the ligand for the Glial Cell Line-Derived Neurotrophic Factor Receptor Alpha-like (GFRAL) to enhance the loss of appetite, whereas the role of the pro-form of NAG-1 has not yet been studied in detail. Essentially, mature NAG-1 expression is significantly elevated under stress conditions, whereas it is expressed at low levels under basal conditions [11]. Our previous studies have demonstrated that, in-vitro, NAG-1 is upregulated at both the transcriptional and translational levels by NSAIDs (nonsteroidal anti-inflammatory drugs) [9] and certain phytochemicals, including resveratrol [12] and quercetin [13]. Interestingly, NAG-1 has been reported to be induced by several anti-obesity and kidney protective compounds including various phytochemicals [12, 14], and its role on anti-obesity has been reported to link to GFRAL binding as a ligand in the brain [15]. Moreover, there are studies reported that NAG-1 has been linked to anti-obesity activity in mice [16] and kidney protection through the protection of renal interstitium and tubular compartment in type 1 and 2 diabetic mouse models [17]. Since NAG-1 could be detected from serum [11], the measurement of NAG-1 in the blood may provide a convenient and approachable tool to determine the risk of obesity and kidney diseases.

This study aimed to clone and characterize the cat *NAG-1* cDNA and explore its potential therapeutic role in obesity and kidney protection. We investigated the effects of anti-obesity and kidney-protective compounds, such as resveratrol and quercetin, on cat *NAG-1* gene expression. Additionally, we examined the renal-protective activity of NAG-1 in cat kidney epithelial cells by evaluating its impact on mitochondrial membrane potential and free radical scavenging under oxidative stress conditions. Our findings suggest that NAG-1 may serve as a promising therapeutic target for preventing obesity and renal failure in cats.

## Results

### Molecular cloning of feline *NAG-1* gene

**Fig. 1:**
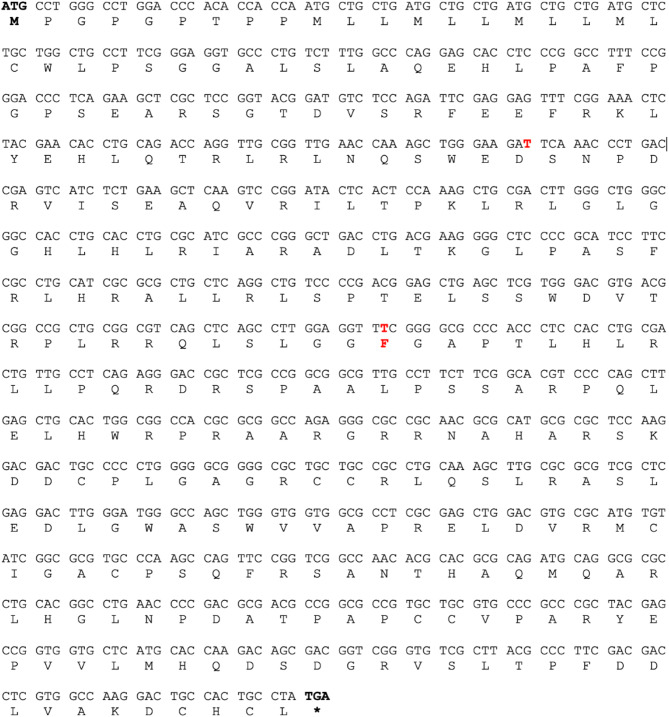
The nucleotide sequence (shown on top) and the deduced amino acid sequence (shown at the bottom) of feline NAG-1 were examined. In the sequences displayed, the portions highlighted in red indicate variations when compared to the reference sequence from the NCBI GenBank (XM_023247515.1)

**Fig. 2:**
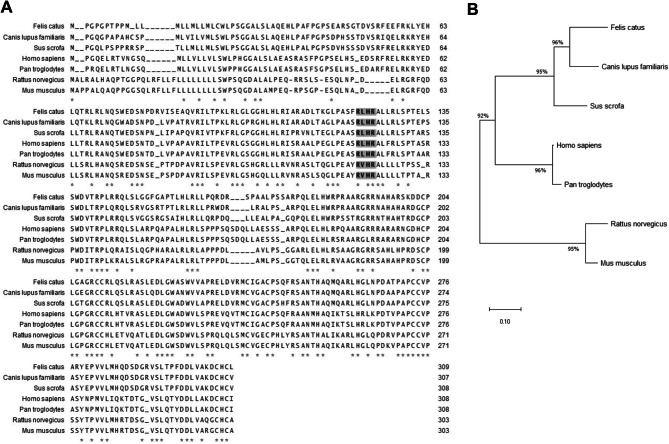
Comparison of NAG-1 protein sequence for various species. (**A**) Multiple alignments of NAG-1 amino acid sequence by species. The positions that are 100% conserved in the alignment are marked with an asterisk (*). A bold letter with a gray background indicates RXXR motifs. (**B**) Phylogenetic tree based on NAG-1 amino acid sequence homology. Except for *Felis catus*, all protein sequence information was obtained by NCBI GenBank (*Canis lupus*, XP_038284083.1; *Sus scrofa*, NP_001167527.1; *Homo sapiens*, NP_004855.2; *Pan troglodytes*, XP_009433302.1; *Rattus norvegicus*, NP_062089.1; *Mus musculus*, NP_001317616.1). The scale bar indicates the number of substitutions per site

The feline uterus tissue was used to isolate total RNA and feline *NAG-1* cDNA was cloned into a TA vector. The primers were designed based on 5’UTR and 3’UTR from *NAG-1* sequence in the GenBank database (NCBI Reference Sequence: XM_045048496.1). The sequence of *NAG-1* obtained from uterus tissue of cat and its deduced amino acid sequences (Fig. 1) were compared to those of other species including dogs, pigs, humans, chimpanzees, rats, and mice. As shown in Fig. 2A, RXXR cleaved site was well conserved in all the species with RL/VHR sequence. In addition, the N-linked glycosylation site in cat *NAG-1* sequence at position 71 of amino acid was conserved in all the species, indicating that glycosylation may contribute to fundamental function of NAG-1 in all species. Phylogenetic tree analysis indicates that cat NAG-1 is closed to dog with 96% similarity (Fig. 2B). Furthermore, the analysis of human, cat, and dog *NAG-1* cDNA sequences with codon usage bias indicates that some codons, such as CTG (Leu), CTC (Leu), GAC (Asp), GCG (Ala) and CGC (Arg), are preferable in the transcripts (Table 1).

**Table 1:** Comparison of NAG-1 codon usage in 3 species (Felis catus, Homo sapiens, Canis lupus familiaris). The feline NAG-1 sequence was obtained by our cloning result. Other nucleic acid sequences were from NCBI Genbank (Canis lupus familiaris, XP_038284083.1; Homo sapiens, NP_004855.2). Fraction refers to the percentage occurrence of synonymous codons encoding NAG-1 amino acids in each species

		Felis catus		Homo sapiens		Canis lupus familiaris	
Amino Acid	Codon	Number	Fraction (%)	Number	Fraction (%)	Number	Fraction (%)
Ala	GCG	15	0.52	11	0.37	12	0.38
	GCA	2	0.07	6	0.2	5	0.16
	GCT	3	0.1	1	0.03	1	0.03
	GCC	9	0.31	12	0.4	14	0.44
Cys	TGT	1	0.1	1	0.11	1	0.1
	TGC	9	0.9	8	0.89	9	0.9
Asp	GAT	2	0.13	3	0.21	1	0.06
	GAC	14	0.88	11	0.79	15	0.94
Glu	GAG	8	0.67	8	0.57	8	1
	GAA	4	0.33	6	0.43	0	0
Phe	TTT	2	0.29	0	0	1	0.33
	TTC	5	0.71	4	1	2	0.67
Gly	GGG	7	0.35	8	0.5	6	0.33
	GGA	5	0.25	2	0.13	4	0.22
	GGT	4	0.2	0	0	3	0.17
	GGC	4	0.2	6	0.38	5	0.28
His	CAT	2	0.17	1	0.08	3	0.21
	CAC	10	0.83	11	0.92	11	0.79
Ile	ATA	1	0.25	2	0.33	2	0.5
	ATT	0	0	1	0.17	0	0
	ATC	3	0.75	3	0.5	2	0.5
Lys	AAG	4	0.8	3	0.6	3	0.75
	AAA	1	0.2	2	0.4	1	0.25
Leu	TTG	7	0.14	6	0.13	6	0.11
	TTA	0	0	1	0.02	0	0
	CTG	27	0.54	25	0.54	24	0.53
	CTA	1	0.02	1	0.02	2	0.04
	CTT	4	0.08	3	0.07	3	0.07
	CTC	11	0.22	10	0.22	11	0.24
Met	ATG	8	1	5	1	6	1
Asn	AAT	0	0	2	0.33	0	0
	AAC	5	1	4	0.67	6	1
Pro	CCG	8	0.28	11	0.41	9	0.29
	CCA	5	0.17	3	0.11	5	0.16
	CCT	7	0.24	1	0.04	7	0.23
	CCC	9	0.31	12	0.44	10	0.32
Gln	CAG	8	0.67	9	0.64	11	0.79
	CAA	4	0.33	5	0.36	3	0.21
Arg	AGG	3	0.09	4	0.11	2	0.05
	AGA	2	0.06	3	0.09	2	0.05
	CGG	9	0.26	12	0.34	11	0.3
	CGA	3	0.09	3	0.09	5	0.14
	CGT	2	0.06	5	0.14	3	0.08
	CGC	16	0.46	8	0.23	14	0.38
Ser	AGT	0	0	1	0.03	2	0.09
	AGC	7	0.29	6	0.21	5	0.22
	TCG	7	0.29	10	0.34	5	0.22
	TCA	2	0.08	2	0.07	2	0.09
	TCT	3	0.13	4	0.14	3	0.13
	TCC	5	0.21	6	0.21	6	0.26
Thr	ACG	7	0.64	6	0.46	5	0.45
	ACA	1	0.09	1	0.08	1	0.09
	ACT	1	0.09	0	0	3	0.27
	ACC	2	0.18	6	0.46	2	0.18
Val	GTG	9	0.75	12	0.8	12	0.75
	GTA	0	0	0	0	2	0.13
	GTT	0	0	0	0	0	0
	GTC	3	0.25	3	0.2	2	0.13
Trp	TGG	6	1	5	1	7	1
Tyr	TAT	0	0	1	0.33	1	0.5
	TAC	2	1	2	0.67	1	0.5
End	TGA	1	1	1	1	1	1
	TAG	0	0	0	0	0	0
	TAA	0	0	0	0	0	0

### Expression and tissue distribution of *fNAG-1*

**Fig. 3:**
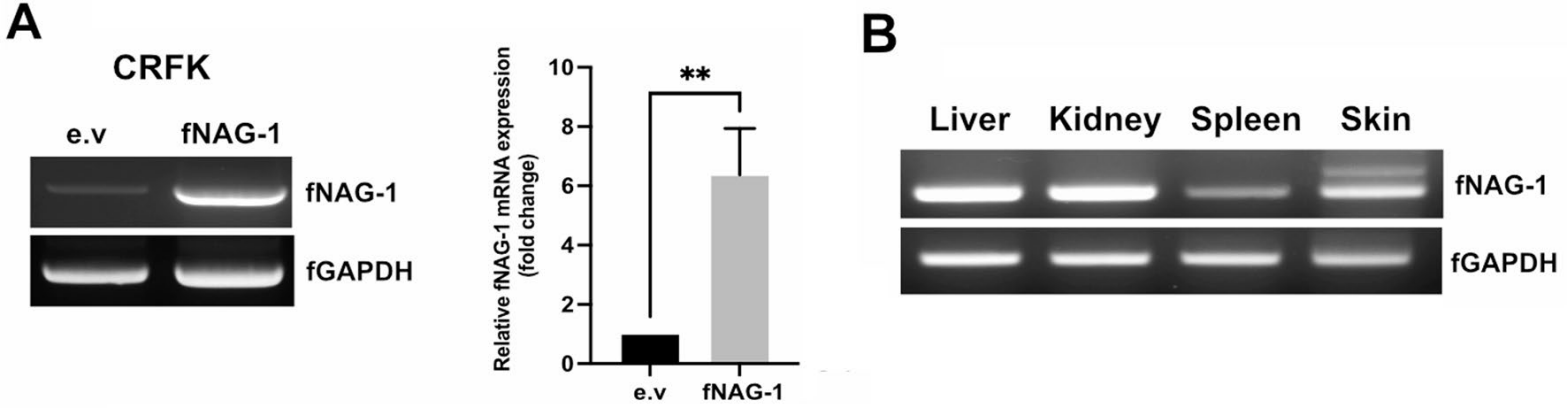
NAG-1 expression in cat tissues. (**A**) Feline NAG-1 expression after transient transfection of feline *NAG-1* cDNA into CRFK cell lines. Feline *NAG-1* and *GAPDH* expression were detected by RT-PCR. The empty vector (e.v) indicates transfection of intact pCDNA3.1 plasmid without any insert cDNA. Results were expressed as mean ± SD (*n* = 3). The statistical analysis was analyzed using student t’s test, **P < 0.01, ***P < 0.001 compared with negative control. (**B**) Expression profiles of *NAG-1* in feline tissues including liver, kidney, spleen, and skin

Feline *NAG-1* was cloned into an expression vector and transfected into cat kidney cells to determine its expression. As shown in Fig. 3A, feline *NAG-1* cDNA was remarkably expressed in CRFK cells. It has been reported that canine NAG-1 expression is distributed in several canine tissues including lung, kidney, and liver [18]. We obtained tissues from the infected cat and investigated NAG-1 tissue distribution. As shown in Fig. 3B, the liver and kidney were the most expressed organ among others, consistent with those seen in mice and dogs [18, 19]. Spleen and skin also expressed *fNAG-1* with relatively lower expression.

### *fNAG-1* induction by the phytochemicals

**Fig. 4:**
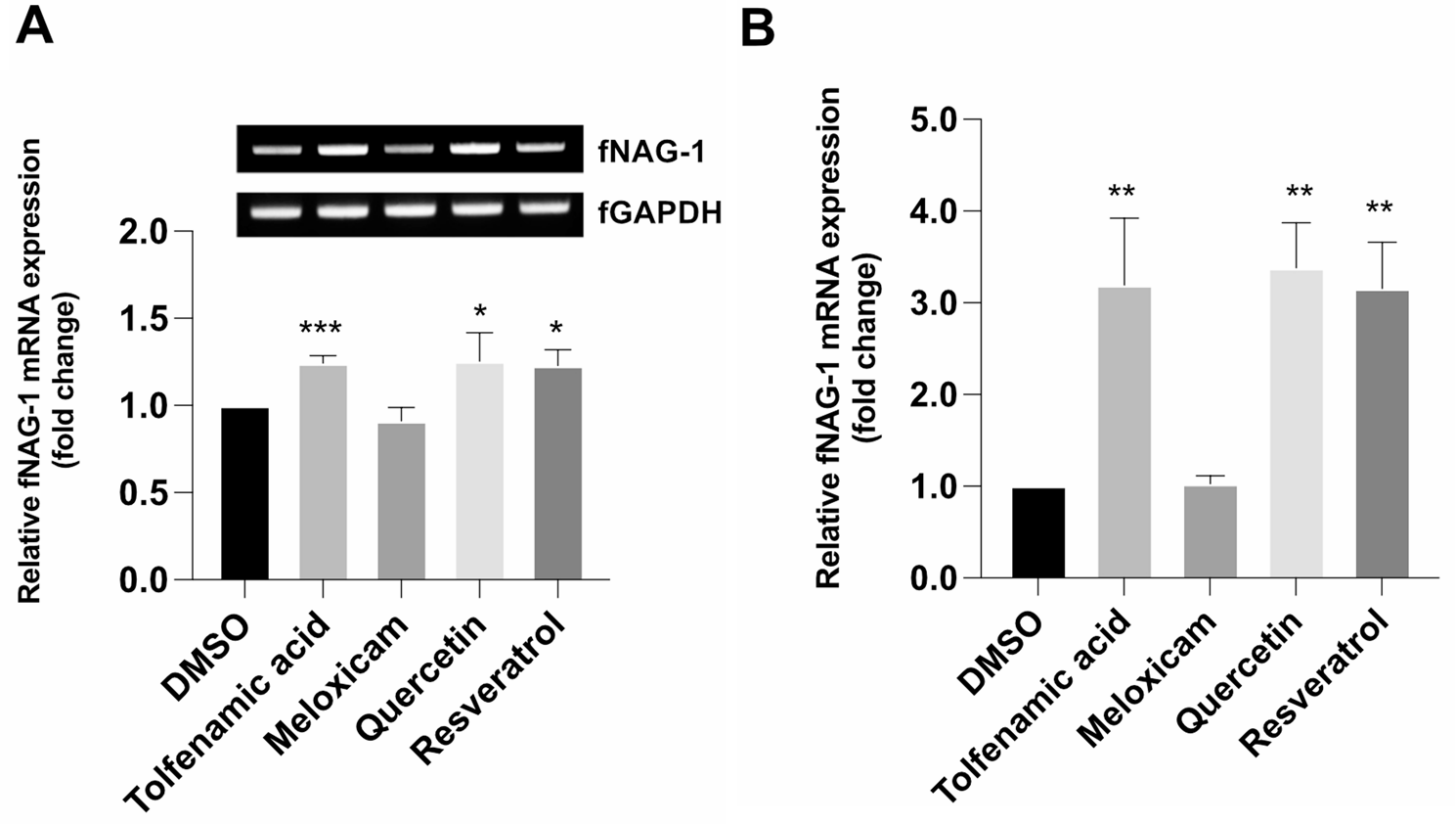
Effects of phytochemicals and NSAIDs in feline kidney cell line. Each compound diluted in serum-free media was treated with CRFK cells and incubated for 24 h. All compounds were used at 50 µM concentration. Feline *NAG-1* and *GAPDH* expression were performed by (**A**) RT-PCR and (**B**) qRT-PCR. Results were expressed as mean ± SD (*n* = 3). The statistical analysis was calculated using One-way ANOVA with Dunnett’s comparison test, *P < 0.05, **P < 0.01, ***P < 0.001 compared with negative control

Quercetin and resveratrol have been reported to protect against kidney injury and to induce anti-obesity effects [20–22]. To determine whether feline NAG-1 is also induced by these phytochemicals as seen in the human *NAG-1* gene, quercetin or resveratrol was treated in CRFK cells and feline NAG-1 expression was measured using two different methods. As shown in Fig. 4A, feline NAG-1 mRNA expression was notably induced by quercetin or resveratrol treatment. NSAIDs also induce NAG-1 expression in human cells [23], and thus tolfenamic acid and meloxicam that are used in cat clinics were treated into CRFK cells. As shown in Fig. 4A, tolfenamic acid also increased NAG-1 expression, whereas meloxicam did not increase NAG-1 expression. Similar results were observed using real-time qPCR (Fig. 4B).

### The biological effect of NAG-1 overexpression in cat kidney epithelial cell lines

**Fig. 5:**
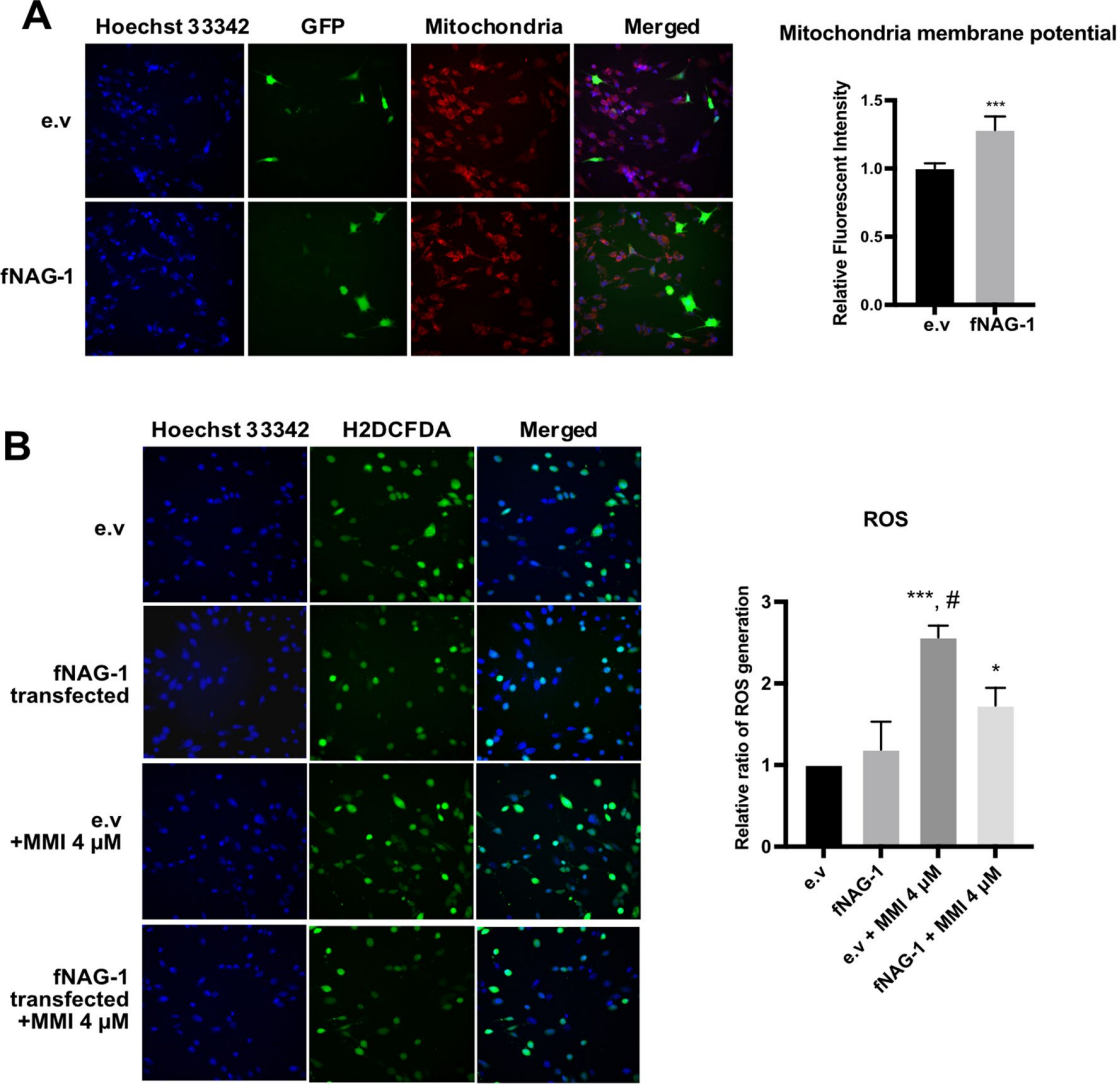
The reno-protection effect of NAG-1 overexpression in cat kidney epithelial cell line. (**A**) The relative fluorescence intensity of mitochondrial membrane potential in empty vector (e.v.) transfected- and feline *NAG-1* transfected cell line. To assess transfection efficiency, all cells, regardless of whether they were transfected with the empty vector (e.v.) or the fNAG-1 expression vector, were additionally co-transfected with a GFP (green fluorescent protein) expression vector. Results are expressed as mean ± SD. The statistical analysis was calculated using student’s t-test method, ***P < 0.001 compared with e.v. transfected cells. (**B**) The relative intensity of H2DCFDA/Hoechst 33,342, and the representative images of the ROS accumulation in MMI induced ROS cells. The cellular ROS scavenging activity was observed in cells transiently transfected with NAG-1 cDNA following exposure to 4 mM MMI for 48 h to induce oxidative stress. While NAG-1 overexpression did not significantly alter basal ROS levels, it demonstrated a protective effect against ROS-induced damage in kidney cells. Images were acquired, and quantitative data were analyzed using high-content screening (HCS) at 200x magnification. The statistical analysis was measured using One-way ANOVA with Tukey’s comparison test (*P < 0.05, ***P < 0.001 compared with e.v transfected cell; ^*#*^*P < 0.05* compared with fNAG-1 transfected cells with 4 µM MMI).

**Fig. 6:**
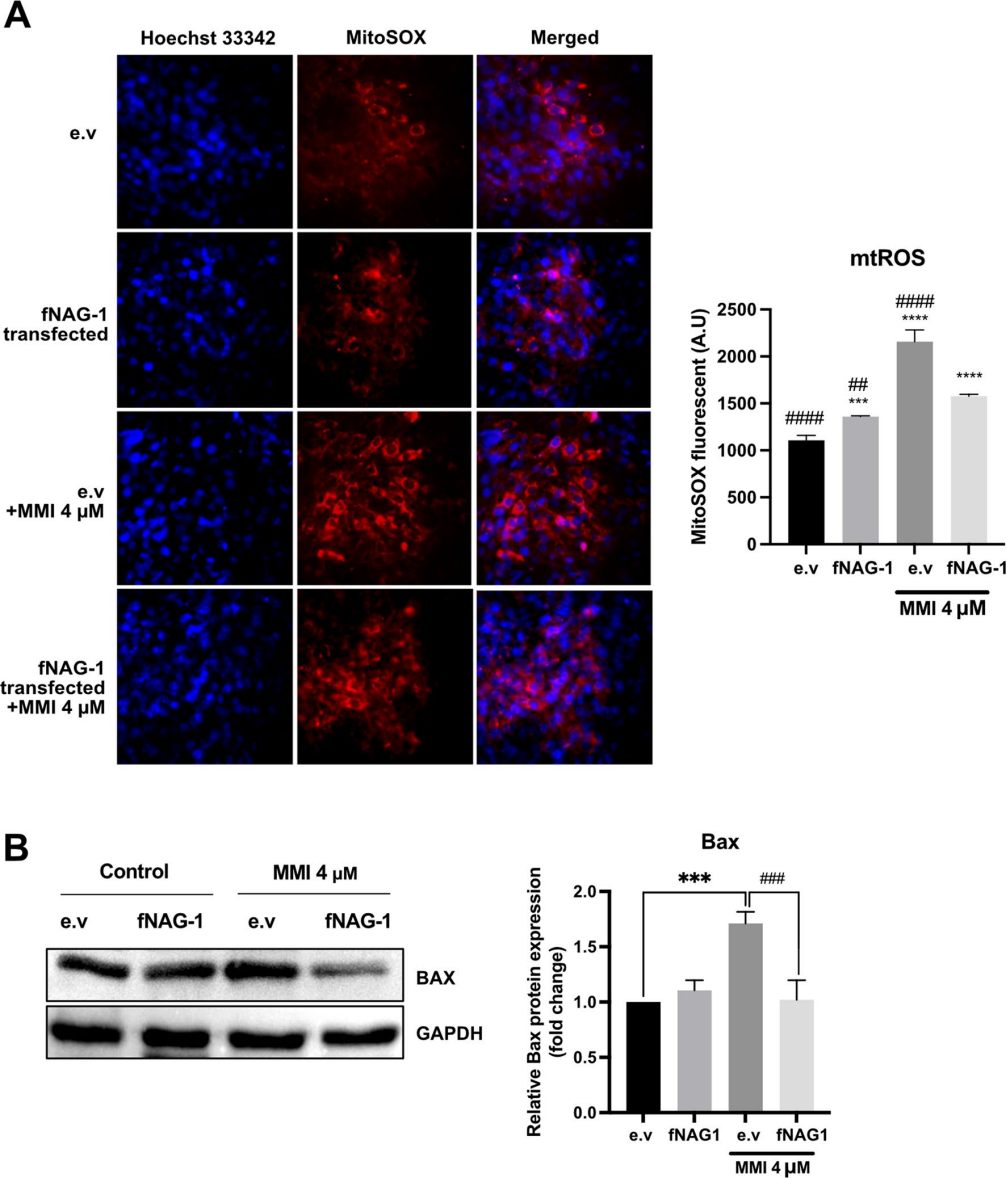
The potential of NAG-1 to reduce the mitochondria ROS generation in CRFK cells. (**A**) The mitochondria ROS assay was performed using mitoSOX, mitochondria superoxide indicator. After transfection CRFK cells with empty vector (e.v.) and feline *NAG-1* plasmid, living cells were stained with mitoSOX for 10 min. Red: MitoSOX. The pictures were captured, and quantitative results were analyzed using high content screening (HCS) with 400x magnification. Results are expressed as mean ± SD. The statistical analysis was calculated using One-way ANOVA with Tukey’s comparison test, ***P < 0.001, ****P < 0.0001 compared with e.v transfected cell; ^*##*^*P < 0.01*, ^*####*^*P < 0.0001* compared with *fNAG-1* transfected cells with 4 µM MMI). (**B**) Attenuation of Bax protein expression in fNAG-1 overexpression cell lines, compared with control, after treating with ROS-induced compound. There was no differential expression of Bax protein in both group in normal condition, whereas NAG-1 transfected cells show lower Bax expression after inducing ROS by 4 µM MMI, compared with their control. Results are expressed as mean ± SD. The statistical analysis was performed using ANOVA with Tukey’s comparison test, ***P < 0.001, compared with e.v transfected cell; ^*###*^*P < 0.001* compared with *fNAG-1* transfected cells with 4 µM MMI

Although NAG-1 expression has been shown to induce apoptosis in cancer cells, it also plays a protective role in several diseases, including cardiovascular disease, Alzheimer’s disease, and kidney failure [17, 24–26]. As shown in Fig. 5A, feline NAG-1 overexpressing cells exhibited increasing mitochondria membrane potential, indicating that NAG-1 enhances CRFK cell viability, probably leading to protective activity in kidney cells. This implies that an increased mitochondrial membrane potential may play a role in protecting kidney cells by enhancing energy production within mitochondria, thereby maintaining cellular health and function. Since ROS is the cause of cellular oxidative stress which could attributes to proteins, lipids, and DNA damage, and utterly leading to apoptotic cell death [27], we determined whether NAG-1 expression reduced ROS generation. The free radicals were induced in renal epithelial cells using methimazole (MMI) at concentration of 4 µM, a concentration equivalent to the maximum plasma levels in oral administration in cats [28]. As shown in Fig. 5B, a significant amount of ROS was generated after MMI treatment; whereas fNAG-1 transfected cell lines exhibited a significant reduction of ROS generation in MMI-induced ROS conditions, compared with those of empty vector (e.v)-transfected cells with 4 µM MMI. In addition, there was no significant effect of NAG-1 on ROS generation in basal conditions, compared fNAG-1 transfected cells with e.v-transfected cells. To determine whether NAG-1 plays role in the reduction of mitochondrial ROS (mtROS), the staining with mitochondria superoxide indicators, mitoSOX were conducted. CRFK cells were transfected with e.v or fNAG1 plasmid and then treated with or without 4 µM MMI. In Fig. 6A, it can be observed that the production of mtROS increased after treatment with 4 µM MMI in both cells transfected with an e.v and cells transfected with fNAG-1. Notably, cells transfected with fNAG-1 but not treated with MMI also showed an elevation in mtROS levels. However, in the presence of MMI, fNAG-1 transfected cells showed significantly lower mtROS generation than e.v-transfected cells. Moreover, the Western blot results (Fig. 6B) also confirmed the potential of fNAG-1 to reduce proapoptotic Bax protein expression compared with e.v transfected cells, in ROS-generated conditions. These results suggest that NAG-1 is capable of protecting kidney cells from free radicals, which can lead to inflammation and fibrosis in the kidney, thereby contributing to impaired kidney function and the progression of CKD.

## Discussion

Due to their tendency to gain excess weight over time, domestic cats are consistently exposed to a range of unfavorable conditions associated with obesity. This chronic exposure can potentially result in metabolic alterations. These metabolic changes often predispose them to develop metabolic diseases, including kidney diseases [29]. CKD is the most prevalent metabolic disorder in cats, with its incidence rising significantly as cats age [30]. Although the direct causal relationship between obesity and CKD in cats has not yet been fully established, the well-documented association in humans suggests a potential connection [31]. This suggests that obesity could act as a contributing or exacerbating factor in the onset and progression of kidney damage in feline patients. Consequently, the development of effective weight management strategies and the identification of novel biomarkers for early detection of obesity in cats could offer significant advantages in mitigating the incidence and prevalence of CKD in this population.

Among the phytochemicals studied in humans, polyphenols such as quercetin and resveratrol have been shown to exert beneficial effects on lipid and energy metabolism as well as body weight control. Furthermore, these compounds have been reported to be associated with kidney protection [32]. Resveratrol demonstrates protective effects against both acute and chronic kidney injuries in humans by its antioxidant properties and its ability to regulate Sirtuin 1 (SIRT1), a modulator of obesity [20]. Therefore, resveratrol holds promise as an additional treatment option for preventing renal injury through SIRT1-dependent or other SIRT1-independent mechanisms. Several studies have also reported the renal protective effect of quercetin. In a rat model of adenine-induced CKD, treatment with quercetin improved renal function by reducing oxidative stress factors, lowering serum levels of fibroblast growth factor 23, and mitigating renal inflammation and tubular damage [21]. While these findings suggest the renal protective effects of quercetin and resveratrol, further investigation is warranted to validate their potential benefits and safety profiles for the prevention and treatment of CKD in clinical settings.

Mitochondria serve as the primary energy-producing organelles, not only in animals but also in plants, and play a crucial role in responding to oxidative stress and inflammation in the kidney. Consequently, mitochondrial dysfunction can contribute to the development and progression of acute kidney injury and CKD. In this study, overexpression of NAG-1 demonstrated significant effects on mitochondrial function and oxidative stress in feline kidney epithelial cells. Under basal conditions, NAG-1 overexpression resulted in a marked increase in mitochondrial membrane potential, indicating enhanced mitochondrial bioenergetics. Additionally, in cells exposed to oxidative stress, NAG-1 overexpression significantly reduced the production of reactive oxygen species (ROS), both at the cellular level and specifically within the mitochondria. Notably, no induction of ROS was detected in cells overexpressing NAG-1 under basal conditions. Additionally, a separate study reported that NAG-1 plays a role in protecting mitochondria in the HT22 neuronal cell line, suggesting that NAG-1 overexpression promotes mitochondrial function by enhancing mitochondrial membrane potential and regulating cell proliferation [33]. On top of that, our previous report also suggested that NAG-1 affects mitochondria membrane potential, leading to the induction of cancer cell apoptosis [13, 14].

Codon usage analysis has been utilized to assess a crucial determinant of mRNA stability, with the presence of rare codons leading to a significant decrease in mRNA stability [34]. The third codon position, specifically guanine and cytosine at the Wobble’s position, also called GC3, has been identified as a potential marker for codon usage bias. Our analysis revealed a bias towards cytosine and guanine usage at the third codon position, with percentages of 77.4% in cats, 75.0% in dogs, and 79.5% in humans (Table 1). It has been reported that genes with a high GC content are more prone to methylation and exhibit more variable expression compared to genes with low GC content [35]. In our study, we observed that NAG-1 genes in these three species, including cats, dogs, and humans, exhibited higher GC content than AT content, suggesting a propensity for methylation and the manifestation of various phenotypic activities. Considering all the above mentioned, several mechanisms may be involved in the regulation of NAG-1 expression including methylation, contributing to its multifunctional activities.

The protein NAG-1 is classified as a cytokine that plays a crucial role in appetite control and body weight regulation, primarily by enhancing thermogenesis and modulating glucose uptake, as demonstrated in mouse models [36]. Additionally, further murine studies have highlighted the protective effects of NAG-1 across various biological contexts, including renal, cardiac, and neuronal cell survival, indicating its promising therapeutic potential for conditions such as kidney injury, myocardial infarction, and Parkinson’s disease [37–39]. Despite the prevalence of obesity and kidney diseases, definitive cures for these conditions are currently lacking. Dietary modifications [40], such as the use of specific food choices, are popular methods employed to prevent and/or slow down the progression of CKD [41] and obesity [42]. In this study, we discovered that NAG-1 expression is induced by two anti-obesity and kidney protective compounds, resveratrol and quercetin. This finding implies that foods containing these compounds may have the potential to prevent obesity or kidney damage in cats.

This study highlights the multifaceted role of NAG-1 in feline obesity and CKD. Given the well-established link between obesity and CKD in both humans and cats, our research aimed to explore the underlying mechanisms connecting these conditions. Notably, NAG-1 exhibits a protective role against mitochondrial dysfunction, a key contributor to CKD pathogenesis, by significantly improving mitochondrial membrane potential and reducing oxidative stress. Additionally, the upregulation of NAG-1 expression by anti-obesity compounds such as resveratrol and quercetin suggests a potential connection between dietary interventions, NAG-1, and the prevention of obesity and CKD. These findings underscore NAG-1 as a promising biomarker for tracking disease progression and a potential therapeutic target for alleviating the effects of obesity on kidney health in feline populations.

To fully understand how NAG-1 regulates mitochondrial function in renal cells and mediates the physiological response to stress, further research is essential. Additional evaluations, such as ATP levels, oxygen consumption rate, and calcium retention capacity, would provide further insights into the potential impact of NAG-1 on mitochondrial function. This study also faced limitations, including the unavailability of appropriate antibodies to detect key proteins in feline samples, preventing the assessment of protein expression and regulation. Moreover, translating the findings to in-vivo experiments in living felines posed significant challenges. Recruiting suitable feline subjects from veterinary clinics was difficult, as was obtaining informed consent from their owners. Despite these obstacles, in-vivo studies are crucial for a deeper understanding of the complex relationship between obesity, CKD, and NAG-1.

In conclusion, our study demonstrated that NAG-1 has a beneficial protective effect on kidney cell function by inducing an increase in mitochondria membrane potential and enhancing ROS scavenging. Furthermore, NAG-1 emerges as a promising target protein with potential implications in the treatment of kidney diseases in cats.

## Materials & methods

### Reagents

Tolfenamic acid was purchased from Cayman Chemical Company (Ann Arbor, MI, USA). Meloxicam was supplied by Tokyo Chemical Industry (Tokyo, Japan) and Quercetin by MO Biomedicals, LLC (Santa Ana, CA, USA). Resveratrol was purchased from Calbiochem (San Diego, CA, USA). These compounds have been used in veterinary clinics and have been shown to increase NAG-1 expression. All compounds were dissolved in dimethyl sulphoxide (DMSO, Biosesang, Gyeonggi-do, South Korea).

### Cell culture

The feline kidney cell line (Crandell-Rees Feline Kidney,CRFK) was purchased from the Korean Cell Line Bank (Seoul, South Korea). CRFK cells were cultured in Dulbecco’s modified Eagle’s medium (DMEM, Welgene, Gyeongsangbuk-do, South Korea) supplemented with 10% FBS (Gibco, Thermo Fisher Scientific, MA, USA) and 1% penicillin/streptomycin (Gibco). All cultured cells were incubated at 37 °C under 5% CO_2_.

### Tissue samples

The uterus sample used for cat cDNA cloning was obtained from a female Korean domestic cat, and the liver, kidney, spleen, and skin tissue samples were obtained from a stray male Korean domestic cat, which were rescued, hospitalized, and euthanized due to terminal illness. Prior to euthanasia, it was confirmed that the animal was in a surgical plane of anesthesia, demonstrating loss of consciousness, reflexes, and response to noxious stimuli. Anesthesia was induced using tiletamine/zolazepam (Zoletil, 10 mg/kg). After the loss of consciousness, euthanasia was conducted via intravenous administration of potassium chloride (85 mg/kg). Euthanasia, due to terminal illness unrelated to the study was performed in accordance with the AVMA Guidelines for the Euthanasia of Animals: 2020 edition. All tissue samples (uterus, liver, kidney, spleen, and skin) were obtained from Seoul National University Veterinary Medical Teaching Hospital (SNU VMTH) and were stored at -80 °C.

### Reverse-transcription polymerase chain reaction (RT-PCR)

The total RNAs from tissues were extracted using the RNeasy Mini Kit (QIAGEN, Hilden, Germany) and the total RNAs from cells were extracted using TRIzol reagent (Ambion, Foster City, CA, USA). The reverse transcription was performed using a Verso cDNA Synthesis Kit (Thermo Fisher Scientific, Waltham, MA, USA), according to the manufacturer’s protocol. The resulting cDNA samples were amplified by PCR using the GoTaq^®^ Green Master Mix (Promega, Madison, WI, USA) and the following specific primers: *Feline*_*NAG-1* (forward) 5’-CAC TCC AAA GCT GCG ACT TG-3’, (reverse) 5’-AAG CGA AAG ACT AGG ACC GC-3’; *Feline GAPDH* (forward) 5’-ACA GTC AAG GCT GAG AAC GG-3’, (reverse) 5’-AAG TCG CAG GAG ACA ACC TG-3’;

The RT-PCR was performed in a G-Storm GS1 Thermal Cycler (G-Storm Ltd., Somerton, UK) with the following cycling steps: initial denaturation at 95 °C for 2 min, followed by 32 cycles of denaturation at 95 °C for 30 s, annealing at 60 °C for 30 s, and elongation at 72 °C for 1 min, and then a final extension at 72 °C for 5 min. The PCR products were analyzed by electrophoresis on 1.5% agarose gels with 5% V/V of DNA staining reagent (NEOgreen, NEO Science, Suwon, South Korea), and visualized using an Alliance Q9 Advanced system (UVTEC CAMBRIDGE, Cambridge, England, UK). The captured images were quantified using ImageJ software 1.53k (National Institutes of Health, Bethesda, MD, USA).

### Quantitative reverse-transcription polymerase chain reaction (RT-qPCR)

The CRFK cell lines were treated with 50 µM of tolfenamic acid, meloxicam, quercetin, and resveratrol. DMSO was used as a negative control. After 24 h, cells were collected and RNA was extracted using the TRIzol™ Reagent (Thermo Fisher Scientific, MA, USA) and synthesized to complementary DNA (cDNA). Ten ng cDNA of each sample were used to perform the DNA amplification using PowerUp™ SYBR™ Green Master Mix (A25741, applied biosystems, Thermo Fisher Scientific, Rockford, IL, USA). The specific primers for RT-qPCR: *Feline_NAG-1* (forward) 5’-CTC CGG TAC GGA TGT CTC CA-3’, (reverse) 5’-TTT GGT TCA ACC GCA ACC TG-3’; *Feline_GAPDH* (forward) 5’-GTC AAG GCT GAG AAC GGG AA-3’, (reverse) 5’-CTC CAT GGT GGT GAA GAC CC-3’. The quantitative data were analyzed using QuantStudio 1 Real-Time PCR system (applied biosystems, Thermo Fisher Scientific, Waltham, MA, USA). The mRNA level of each gene was normalized to that of GAPDH. The relative gene expression was calculated using the comparative Ct method (ΔΔCT Method) as previously described [16].

### Generation of feline NAG-1 expression plasmid

To amplify the feline NAG-1 coding sequence, total RNA was extracted from cat uterus tissue by using an RNeasy Mini Kit. Total RNA (1 µg) was reverse transcribed into cDNA using a Verso cDNA synthesis kit (AB1453B, Thermo Fisher, IL, USA) using a MiniAmp Plus Thermal Cycler (Applied Biosystems, Waltham, MA, USA). RT-PCR was performed using GoTaq^®^ Green Master Mix with the following primers: *Feline NAG-1*_full length (forward) 5’-GCA CAG GCA TGC CTG GGC CTG G-3’, (reverse) 5’-CTA GGA CCG CTC ATA GGC AGT GGC-3’. The thermal cycling conditions consisted of an initial denaturation at 95 °C for 2 min, followed by 40 cycles of 95 °C for 30 s, 65.5 °C for 45 s, and 74 °C for 2 min, with a final extension at 74 °C for 5 min. The PCR product was gel-purified using QIAquick Gel Extraction Kit (QIAGEN, Hilden, Germany), the amplified feline NAG-1 coding DNA was cloned into pTOP V2 vector by using TOPcloner TA core kit (Enzynomics, Daejeon, Korea), according to the manufacturer’s protocol. The resulting recombinant plasmids were isolated and verified by DNA sequencing. To generate a feline NAG-1 expression clone, the coding sequence of feline NAG-1 in pTOP V2 vector and pCDNA3.1 vector were digested by EcoRI restriction enzyme. Then, the EcoRI-digested pCDNA3.1 vector was dephosphorylated by using Calf Intestinal Alkaline Phosphatase (Invitrogen, Carlsbad, CA, USA) followed by ligation with EcoRI-digested feline NAG-1 coding sequence using T4 DNA ligase (Enzynomics, Daejeon, Korea) at 4 °C, overnight. After transformation and DNA extraction, the ligation products were verified by both restriction enzyme digestion and DNA sequencing.

### Sequence analysis

For DNA sequencing, the forward (T7 promotor) primer and reverse (BGH-R) primer were used for determining the sequence of nucleotides. The sequences were aligned using the Needleman-Wunsch algorithm global alignment (https://blast.ncbi.nlm.nih.gov/Blast.cgi). The deduced amino acid sequence of the feline NAG-1 protein was generated by the Expasy translate tool (https://web.expasy.org/translate/) which is operated by the Swiss Institute of Bioinformatics. The feline NAG-1 protein sequence result was compared with those of other species from the NCBI GenBank database and multiple sequence alignment was performed by the T-Coffee program [43] (http://tcoffee.crg.cat/apps/tcoffee/index.html). The NAG-1 codon usages of each species were analyzed online on the Sequence Manipulation Suite [44] (https://www.bioinformatics.org/sms2/codon_usage.html).

### Transient transfection of the NAG-1 expression vector into cell lines

The completed feline NAG-1 expression vectors were transfected into CRFK cell line using Polyjet™ in-vitro DNA transfection reagent (SignaGen, Frederick, MD, USA). Briefly, 1 × 10^6^ cells/dish were seeded into 6-cm dish and further incubated for 18 h. The complex of DNA and transfection reagent (2.5 µg of DNA plasmid and 7.5 µL of transfection reagent) in serum free media was placed into CRFK cells. After 12 h of transfection, the cell culture media was then substituted with fresh complete media and further incubate for 6 h. According to the above method, the RNA was extracted from transfected cells and PCR was performed for confirming feline NAG-1 expression. The empty pCDNA3.1 vector was used as a negative control.

### Compound treatment

CRFK cells were seeded into 60 mm dishes and grown to 70% confluency. After 24 h of treatment with the compound in serum-free media, cells were harvested using TRIzol reagent and PCR was performed. The final concentration of 50 µM tolfenamic acid, meloxicam, quercetin, and resveratrol, dissolved in DMSO, were used for treatment. DMSO treatment was used as a negative control.

### Mitochondrial membrane potential detection

Mitochondrial membrane potential analysis by high-content screening was previously reported [45]. Briefly, CRFK cells were seeded at 1 × 10^5^ cells/well into 48 well-plates, and further incubated for 18 h. The cells were stained with 10 µg/ml of Hoechst 33,342 (Sigma-Aldrich, St. Louis, MO, USA) and 100 nM of MitoTracker Orange CMTMRos (Thermo Fisher Scientific, Waltham, MA, USA) for 15 min at 37 °C and then washing with PBS for 4 times. The fluorescence intensity was measured using the CX7 LZR HCS platform (Thermo Fisher Scientific, Waltham, MA, USA), with same exposure times.

### Reactive oxygen species (ROS) assay

The CRFK cells were seeded at 1 × 10^5^ cells/well into 48 well-plate and further incubated for 18 h. After transient transfection with fNAG-1 plasmid, cells were treated with 4 µM of methimazole (MMI, #46429, Supelco., PA, USA) for 48 h to induce ROS generation [46]. Then, cells were stained with 10 µg/ml of 2’,7’-dichlorofluorescin diacetate (DCFDA, #D6883, Sigma Aldrich) and 1 µg/mL Hoechst 33,342 (Sigma-Aldrich, St. Louis, MO, USA) for 30 min at 37 °C. After washing with PBS 4 times, the cells were visualized, and the intensity was measured using the CX7 LZR HCS platform.

### Mitochondrial reactive oxygen species (mtROS) assay

The CRFK cells were seeded at 1 × 10^5^ cells/well into 48 well-plates. After incubation for 24 h, cells were transfected with the *fNAG-1* plasmid or e.v, and then treated with 4 µM of methimazole for 48 h, followed by staining cells with 5 µM MitoSOX™ Mitochondrial Superoxide Indicators (M36008, Invitrogen) and 1 µg/mL Hoechst 33,342 for 10 min at 37 °C. After washing with 1xPBS four times, the picture of cells was captured, and the fluorescent intensity was measured by the CX7 LZR HCS platform.

### Immunoblotting

After cells were washed with PBS two times, the cells were lysed with a RIPA buffer (#BRA0500, Biomax, Gyeonggi-do, Korea) containing universal protease inhibitor cocktail (#BPI0001, Biomax). The concentration of proteins was quantified using the Pierce™ BCA protein assay kit (Thermo Scientific, Abbott Park, IL, USA). Total proteins (50 µg) of each sample were separated using 10% sodium dodecyl sulfate-polyacrylamide gel electrophoresis (SDS-PAGE) and transferred to a PVDF membrane. Following, the membrane was blocked with 5% BSA for one hour at RT, and the specific primary antibodies, rabbit anti-Bax (dilution of 1:1000, D2E11, #5023, Cell Signaling, MA, USA), was used for incubating the membrane at 4 °C for overnight. After washing using TBST buffer (containing 0.1% of Tween20), HRP-conjugated IgG secondary antibody (1:5000 dilution, #31460, Thermo Scientific) in 5% skim milk was used for incubate the membrane for 2 h at RT. The anti-GAPDH HRP-conjugated (dilution of 1:2000, G9, #sc-365062, Santa Cruz, TX, USA) was used to determine the house keeping protein, GAPDH. Before membranes were visualized using Alliance Q9 mini (Cambridge, UK) and quantified using ImageJ software, the blotted membrane was developed using ECL substrate (Pierce™ ECL Western Blotting Substrate, 32106, Thermo Scientific).

### Statistical analysis

All experiments were performed on at least three independent experiments. Statistical analysis was performed using GraphPad Prism 9 software (version 9.5.0). Values are expressed as mean ± SD and data were analyzed using a student’s *t*-test or One-way ANOVA followed by Tukey’s multiple comparisons test. P-value < 0.05 was considered statistically significant.

## Electronic supplementary material

Below is the link to the electronic supplementary material.

Supplementary Material 1

## Data Availability

The datasets analyzed during the current study are available in the GenBank repository, OQ973470. The other data that support the findings of this study are available on request from the corresponding author on reasonable request.

## Abbreviations

3’UTR: 3’ Untranslated Region
5’UTR: 5’ Untranslated Region
cDNA: Complementary DNA
CKD: Chronic kidney disease
CRFK: Crandell-Rees Feline Kidney
DMEM: Dulbecco’s Modified Eagle Medium
DMSO: Dimethyl sulfoxide
e.v: Empty Vector
FBS: Fetal bovine serum
FGF23: Fibroblast growth factor-23
fNAG-1: Feline NAG-1
GDF15: Growth differentiation factor 15
MMI: Methimazole
mtROS: Mitochondrial reactive oxygen species
NAG-1: Nonsteroidal anti-inflammatory drug (NSAID)-activated gene-1
NSAIDs: Non-steroidal anti-inflammatory drugs
PVDF: Polyvinylidene fluoride
ROS: Reactive oxygen species
RT-PCR: Reverse transcription polymerase chain reaction
RT-qPCR: Quantitative reverse transcription polymerase chain reaction
SIRT1: Sirtuin 1
TBST: Tris Buffered Saline with Tween 20
