# Supplementary material for: Characterization of feline nonsteroidal anti-inflammatory drug activated gene-1 (fNAG-1) and its protective function in kidney cells
Source: BMC Vet Res. 2025 May 21;21:364. doi: 10.1186/s12917-025-04781-1 (PMC12093827; doi:10.1186/s12917-025-04781-1)

Supplementary information

Supplementary Figure 1. RT-PCR results of human NAG1 (Product size = 169 bp) and those of their GAPDH (product size = 453 bp) in 293T cell line.

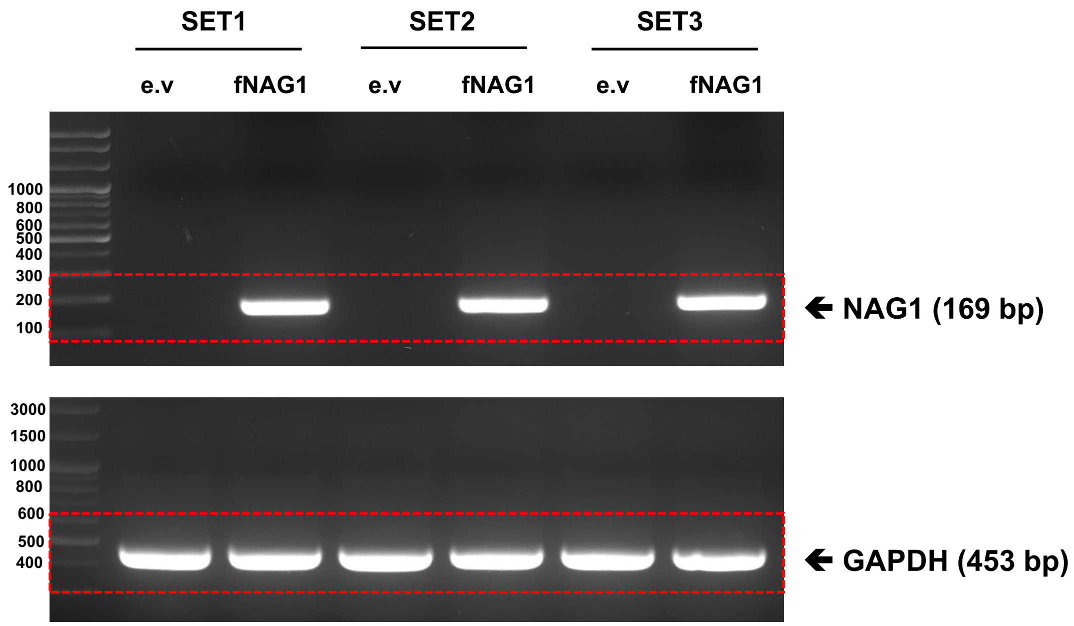

Supplementary Figure 2. RT-PCR results of feline NAG1 (Product size = 678 bp) and those of their GAPDH (product size = 683 bp) in CRFK cell line.

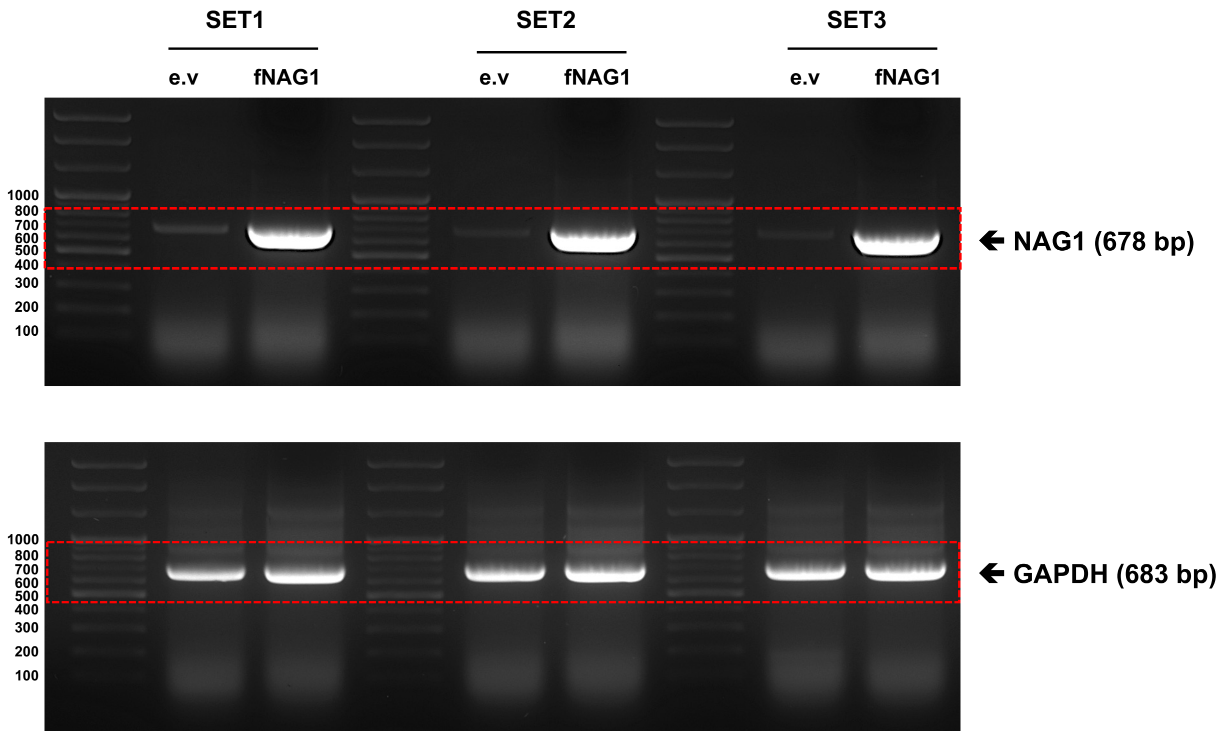

Supplementary Figure 3. Western blot results of Bax (MW = 20 kDA) and those of their GAPDH (MW = 36 kDA)

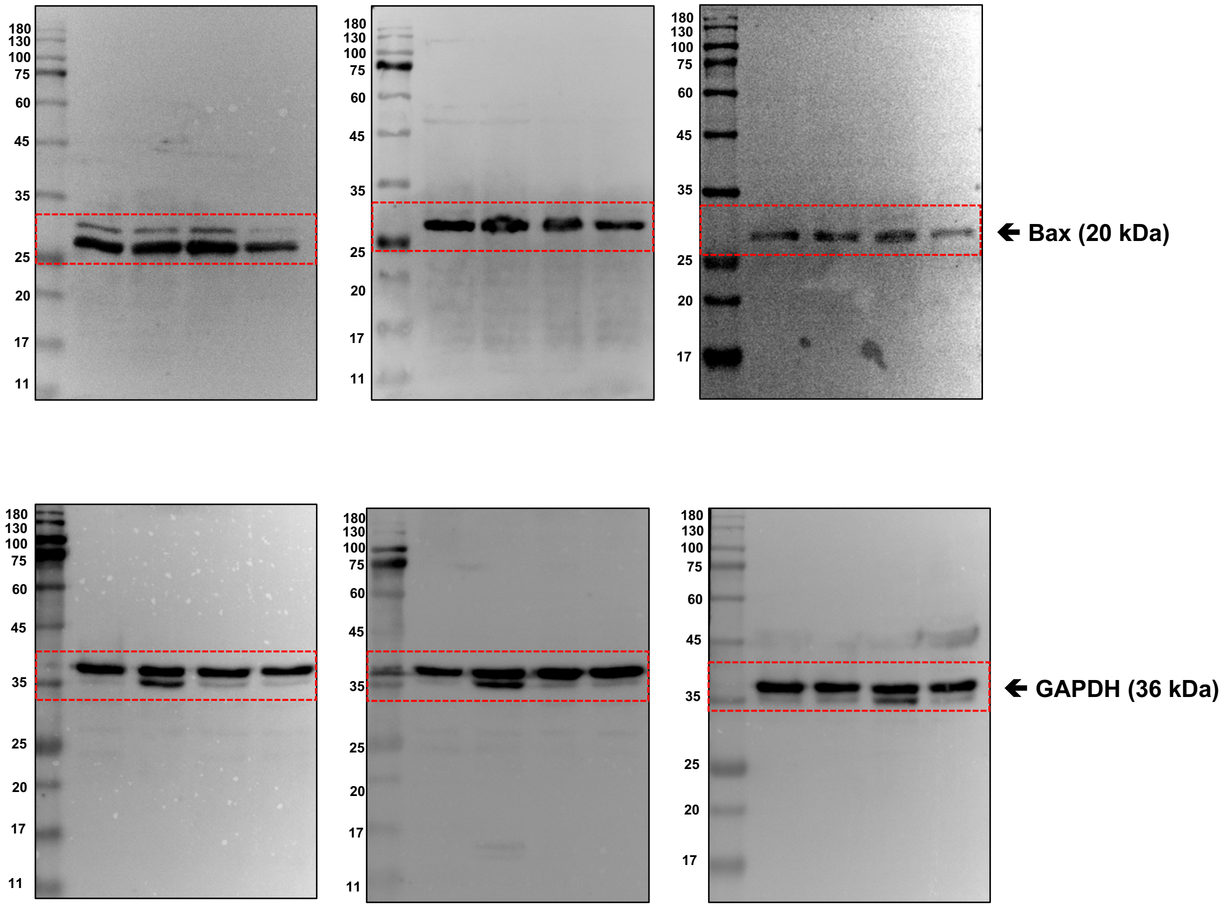

Supplement: Supplementary file 1 — Supplementary Material 1 [file 12917_2025_4781_MOESM1_ESM.pdf]
